# Time- and Zinc-Related Changes in Biomechanical Properties of Human Colorectal Cancer Cells Examined by Atomic Force Microscopy

**DOI:** 10.3390/biology9120468

**Published:** 2020-12-14

**Authors:** Maria Maares, Claudia Keil, Leif Löher, Andreas Weber, Amsatou Andorfer-Sarr, Hajo Haase, Jagoba Iturri, José L. Toca-Herrera

**Affiliations:** 1 Chair of Food Chemistry and Toxicology, Technische Universität Berlin, Straße des 17. Juni 135, 10623 Berlin, Germany; c.keil@tu-berlin.de (C.K.); leif.loeher@gmail.com (L.L.); haase@tu-berlin.de (H.H.); 2 Institute for Biophysics, Department of Nanobiotechnology, BOKU University for Natural Resources and Life Sciences, Muthgasse 11 (Simon Zeisel Haus), 1190 Vienna, Austria; andreas.weber@boku.ac.at (A.W.); amsatou.andorfer-sarr@boku.ac.at (A.A.-S.); jose.toca-herrera@boku.ac.at (J.L.T.-H.)

**Keywords:** colorectal cancer cells, zinc supply, atomic force microscopy, cell mechanics, cell proliferation

## Abstract

**Simple Summary:**

We aimed to study how cellular zinc status (adequate vs. deficiency), closely related to colorectal cancer, does affect the nanomechanical properties of cell lines HT-29 and HT-29-MTX during their early proliferation (24–96 h). These properties and their variations can be characterized by means of Atomic Force Microscopy (AFM), a technique that allows perpendicular indentation of cells with a sharp nanometric tip, under controlled speed and load, while recording the real time variation of tip-to-cell interacting forces on approach, contact, and retraction segments. From each of these sections, complete information about the respective elastic modulus, relaxation behavior, and adhesion is extracted, thus identifying cell line- and zinc-related nanomechanical fingerprints. Our results show how the impact of zinc deficiency on the mechanical response of the cells underlines the relevance of monitoring the nutritional zinc status of tumor samples when analyzing cancerous tissues or single cells with AFM, particularly regarding the development and validation of biomechanical fingerprints as diagnostic markers for cancer.

**Abstract:**

Monitoring biomechanics of cells or tissue biopsies employing atomic force microscopy (AFM) offers great potential to identify diagnostic biomarkers for diseases, such as colorectal cancer (CRC). Data on the mechanical properties of CRC cells, however, are still scarce. There is strong evidence that the individual zinc status is related to CRC risk. Thus, this study investigates the impact of differing zinc supply on the mechanical response of the in vitro CRC cell lines HT-29 and HT-29-MTX during their early proliferation (24–96 h) by measuring elastic modulus, relaxation behavior, and adhesion factors using AFM. The differing zinc supply severely altered the proliferation of these cells and markedly affected their mechanical properties. Accordingly, zinc deficiency led to softer cells, quantitatively described by 20–30% lower Young’s modulus, which was also reflected by relevant changes in adhesion and rupture event distribution compared to those measured for the respective zinc-adequate cultured cells. These results demonstrate that the nutritional zinc supply severely affects the nanomechanical response of CRC cell lines and highlights the relevance of monitoring the zinc content of cancerous cells or biopsies when studying their biomechanics with AFM in the future.

## 1. Introduction

The study and characterization of the mechanical properties in cells have sparked great interest recently, particularly regarding their contribution to cell structure and activity [1,2,3]. A detailed description of such properties can confirm regular cell functioning. Mechanical properties have become a good detection tool for abnormalities caused by several diseases, with cancer being its maximum exponent [4,5,6]. Several studies on breast, lung, and bladder cancer cells provide relevant examples of the information obtained by the characterization of their respective mechanics [7]. An already established cancerous-like behavior is reflected in the form of tissue stiffening for most cancer types, although exceptions to this rule have been described as well [7,8,9]. Moreover, the adhesion capability of individual cancer cells can be affected by tumor progression [10,11,12], which could be explained by shifting of the membrane potential toward a depolarized state [13], or through (over)expression of diverse membrane receptors [14]. These prognostic markers can be applied as targets for tumor imaging and pharmaceutical treatments [15,16]. In fact, monitoring of biomechanical factors in diagnostic research for diseases offers great potential to find new diagnostic factors, particularly in the development of biomarkers to identify cancerous cells and distinguish between differences involving normal tissues at an early stage of the disease [5,7,9]. The application of atomic force microscopy (AFM) in force spectroscopy mode seems to be quite promising, as it represents a powerful tool to relate mechanical changes to cellular function and structure [8]. For such a purpose, nanomechanical properties of tumors, including the elasticity and deformability of cancerous cells, are screened via AFM to identify and validate characteristic fingerprints of cancerous tissue sections and biopsies for future use as diagnostic markers [9,17]. This demands a comprehensive collection of data on the biomechanics of single (cancerous) cells, which has been mainly generated by studying different in vitro cultured carcinoma cell lines [8]. In fact, these cell lines are often used to examine concentration and time-dependent exposure of cancerous cells to different pharmacological or bioactive compounds via AFM [18,19]. Biomechanical properties have already been studied for various tissues [20], among them the gastrointestinal tract as well as the colon [21], though this technique has been predominantly applied to study non-pathological tissues [22,23], and less to differentiate between healthy and deteriorated, abnormal tissue from colon biopsies. Data on biomechanical properties of colon carcinoma cell lines measured with AFM are scarce, both regarding their reaction before and after treatment with nutritive as well as pharmacological substances [24,25].

There is epidemiological and genetic evidence that several types of cancer can be prevented through lifestyle and appropriate diet modifications [26]. Micronutrients, particularly the essential trace element zinc, which is a key constituent and co-factor of numerous proteins [27], are discussed to be of particular importance for the host defense against cancer initiation and progression and to potentially function as chemopreventives [28,29,30]. Accordingly, an appropriate quality and quantity of these nutrients has to be provided as part of a person’s diet [31,32]. According to the GLOBOCAN data, 18.1 million incidences of new cancer cases have been reported in 2018 [33], with colorectal cancer (CRC) being the third most deadly and fourth most commonly diagnosed cancer in the world [34]. Even though data on the reduction of CRC in association with dietary zinc intake is not yet fully conclusive from currently published prospective studies [35,36] and comprehensive retrospective studies correlating the individual zinc status and progression of CRC are scarce [37], there is strong evidence that the systemic zinc status is related to the CRC risk. This is supported by the fact that the plasma copper to zinc ratio is currently discussed as a pre-diagnostic marker for CRC [36]. Furthermore, expression of the zinc transporting proteins solute carrier (SLC)30/zinc transporter (ZnTs) and SLC39A/Zrt-/Irt-like proteins (ZIPs) is deregulated in human CRC tissues as well as CRC cell lines compared to healthy colonic mucosa, indicating that zinc homeostasis in CRC is altered on the (sub)cellular level [38]. Critical changes of zinc homeostasis and zinc transporter expression were reported in various types of cancer [39,40], impacting epithelial-mesenchymal transition (EMT) in tumors [41,42] as well as cancer proliferation and metastasis [43,44]. Yet, these processes are not in general comparable between different cancer types and tissue zinc level alteration is also highly cancer-specific [45,46], hence in how much altered zinc levels impact EMT and cancer metastasis in CRC is still unclear. Animal studies suggest that zinc deficiency contributes to the development and progression of CRC, linking low zinc levels in intestinal tissues to the development of pre-neoplastic lesions and colon carcinogenesis in rats [47]. Zinc deficiency in vivo is associated with enhanced production of reactive oxygen species (ROS), increased oxidative stress, as well as perturbed antioxidative protection, impaired DNA-repair, and DNA-response mechanisms by affecting the expression of the tumor suppressor protein p53 and several important transcription factors, such as nuclear factor ‘κ-light-chain-enhancer’ of activated B-cells (NF-κB) and activator protein (AP)-1, leading to increased DNA-damage, mutations, genomic instabilities, and elevated inflammation, which consequentially elevates the risk of cancer [28,29,48]. In vitro studies with human CRC-derived cell lines observed alterations in junctional and cytoskeleton proteins during zinc deficiency [49]. However, to what extent this also affects the biomechanics of these cells [24,50] and whether this might be of importance regarding the development of diagnostic biomarkers for cancer utilizing AFM [51] remains to be investigated.

Regarding the overall aim to identify and validate characteristic nanomechanical fingerprints for tumor diagnostics, the effect of the cellular zinc status on the mechanics of CRC needs to be included when collecting required data on the cell mechanical properties of individual cancerous cells. As data on the mechanics of CRC on the nanoscale are lacking and the impact of the micronutrient zinc is still unknown, this study aims to examine the biomechanics of two CRC cell lines and the influence of the zinc availability on these parameters via AFM. For this, proliferating CRC cell lines HT-29 and HT-29-MTX were subjected to zinc deficiency (zinc-deficient, ZD) and compared to zinc-adequate (ZA) cultured cells. The choice of these two CRC cell lines is based on the fact that HT-29 colonocytes are widely used as a tumor model to study colorectal cancer, are sensitive to chemotherapeutic drugs and grow as undifferentiated, heterogeneous, and unpolarized epithelial cells forming multi-layers when cultured under standard conditions [52,53]. Yet, changing these culture conditions or treating HT-29 with inducers, such as butyrate, lead to cell differentiation and formation of polarized absorptive intestinal epithelial cells after culturing for extended time periods [52,54]. HT-29-MTX cells represent a stable subclone derived from proliferating HT-29 treated with methotrexate (MTX) and isolated through selective-pressure [55,56]. This homogenous cell line resembles epithelial colonocytes in their proliferating state and differentiates into mature mucin-producing human goblet cells when cultured for 14–21 days [57]. The latter has also been their main application in research so far, while cellular and biomechanical properties of proliferating HT-29-MTX have not yet been studied. Both cell lines express the main gastrointestinal zinc transporters, are well characterized regarding their (sub)cellular zinc homeostasis and can both be experimentally subjected to a zinc dyshomeostasis and deficiency [58,59,60,61]. This enabled us to gain insights into time- and zinc-dependent changes of the biomechanics of CRC cells.

## 2. Materials and Methods

### 2.1. Sample Preparation

Borosilicate circular cover glasses (diameter: 24 mm, thickness: 0.08–0.12 mm, Menzel Gläser) were sonicated in ethanol, dried under N_2_, and cleaned by using oxygen plasma (Gala Instrumente, Bad Schwalbach, Germany) for 60 s, to be subsequently taken to the cell culture lab.

### 2.2. Preparation of Zinc-Deficient Medium

Dulbecco’s Modified Eagle’s Medium (DMEM) (PAN-Biotech, Aidenbach, Germany), supplemented with 10% fetal bovine serum (FBS) (CCPro, Oberdorla, Germany), 100 U/mL penicillin, 100 µg/mL streptomycin, and 1% non-essential amino acids (NEAA) (Sigma Aldrich, Munich, Germany) (complete DMEM), was treated with Chelex^®^ 100 Resin (Bio-Rad, Hercules, CA, USA, 50 g/L medium) for 24 h in order to remove zinc from the medium, and was then sterile filtered (0.2 µm cut off filter, Sigma Aldrich, Munich, Germany) as reported [58].

### 2.3. Cell Culture

CRC cell lines HT-29-MTX-E12 [56] and HT-29 [53] were obtained from the European Collection of Authenticated Cell Cultures (ECACC, Porton Down, UK). Cells were cultivated in complete DMEM at 37 °C and 5% CO_2_. Cells (4 × 10^4^) were transferred on plasma pre-cleaned glass slides, and incubated for 24, 48, 72 or 96 h at 37 °C with either zinc-adequate (ZA, total zinc 3 µM) or zinc-deficient (ZD, zinc content < LOQ [58]) medium. After the corresponding incubation, Leibovitz’s L-15 medium without FBS (zinc content < LOQ) was added and the pre-confluent and proliferating cells were directly imaged by using optical microscopy (Zeiss Axio Observer Z1, Jena, Germany).

### 2.4. Cell Proliferation

HT-29-MTX and HT-29 were cultured for 24–96 h in 96 well plates and cell growth and proliferation were investigated by measuring cellular dehydrogenase activity using water soluble tetrazolium (WST)-8 (Sigma Aldrich, Munich, Germany), and total cellular protein via sulforhodamine B (SRB)-assay (Sigma Aldrich, Munich, Germany), as described elsewhere [62].

### 2.5. Atomic Force Microscopy (AFM)

AFM measurements were performed by using a Nanowizard 3 (JPK Instruments, Berlin, Germany) in Force Spectroscopy mode mounted on an inverted optical microscope (Axio Observer Z1, Zeiss, Germany). Temperature of the experiments was kept at 37 °C by using the commercial BioCell™ coverslip-based liquid cell (JPK). The piezo range in Z axis could be extended to 100 µm through employment of a CellHesion© module add-on. Silicon nitride cantilevers DNP-S10 (Bruker, Billerica, MA, USA) with a pyramidal tip and an average indenter diameter of 22 nm were chosen for sample probing. These were cleaned by using oxygen plasma, rinsed with Ethanol, and gently dried with N_2_ prior to their use. The spring constant of the cantilevers (nominal: 0.12 N/m) was calibrated before experiments using thermal noise tuning. A minimum of three samples, 2–3 locations each, were employed for the corresponding conditions, ensuring measurement of a sufficiently high number of cells.

**Figure 1:**
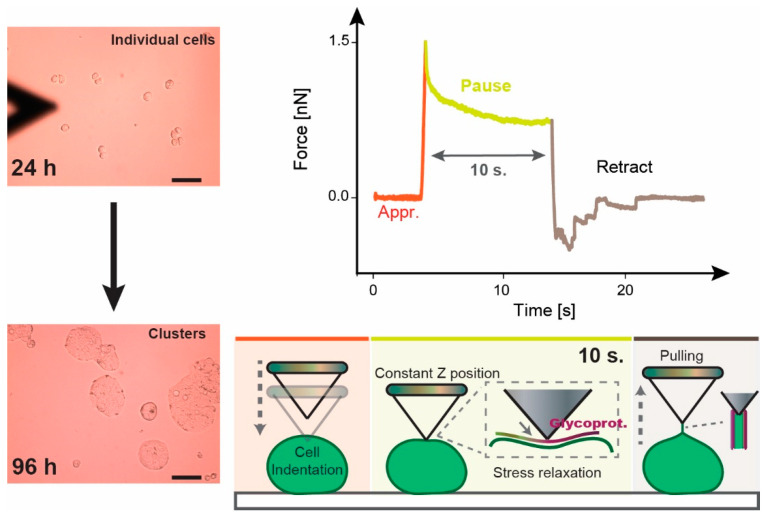
Graphical Scheme. Left: microscopy image of the evolution of HT-29-MTX cells with time. Scale bar indicates 50 µm. Right: example of a stress relaxation experiment (Force vs. time) monitored using the cell indentation process by following the steps in the sketch below.

Measurements were then carried out in liquid (Leibovitz’s L15), keeping both the rate (approaching and pulling speed of the cantilever, 5 µm/s) and the loading force (1.5 nN) constant. The influence of both factors on cell mechanics was considered, as was recently described by Weber et al. [63]. In addition, the stress relaxation assays on the cells were measured by keeping the Z position of the cantilever constant for 10 s (see the scheme in Figure 1).

### 2.6. Data Analysis

The recorded force-distance and force-time curves were analyzed using JPK-Software (JPK, Berlin, Germany). The so-obtained data were plotted with OriginPro 9. Optical microscopy images were treated by using Zen Blue Edition software (Zeiss, Germany), which also allowed determination of the cell body area. Statistical (ANOVA, Student *t*-test), and mathematical analyses were performed using OriginPro 9 (OriginLab Corporation, Northampton, MA, USA). Normally distributed data sets were evaluated by using Gaussian fitting, calculation of mean value, and the standard error of the mean. Mechanics-related factors followed the following protocols:

#### 2.6.1. Elastic Modulus E

For calculation of the Young’s Modulus E, the Sneddon extension of the Hertz model for four-sided pyramidal indenters (Equation (1)) was used in the data analysis software:

(1) $$F=\frac{E}{1-\upsilon }\frac{\tan\left(\alpha \right)}{\sqrt{2}}\delta^{2}$$

where E is the Young’s Modulus, ν is the Poisson’s ratio (set to 0.5 assuming cells are incompressible), α is the face angle of the pyramid (22°), and δ is the indentation. An indentation of 350 nm (corresponding to less than 10% of the cell height) was used to calculate the Young’s Modulus.

#### 2.6.2. Stress Relaxation

Evaluation of relaxation mechanics was performed by considering a parallel arrangement of viscoelastic components, which was fitted by using a form of double-exponential force decay behavior (Equation (2)):

(2) $$F\left(t\right)=A_{1}{\;e}^{-\frac{\left(t-t_{0}\right)}{\tau_{1}}}+{\;A}_{2}{\;e}^{-\frac{\left(t-t_{0}\right)}{\tau_{2}}}$$

with A_1_ and A_2_ the decay amplitudes, and τ_1_ and τ_2_ as the respective relaxation time of the individual viscoelastic constituent (membrane, cytoskeleton, etc.). Figure 1 shows a representative force vs. time plot as obtained for the pause segment.

#### 2.6.3. Adhesion Factors

**Figure 2:**
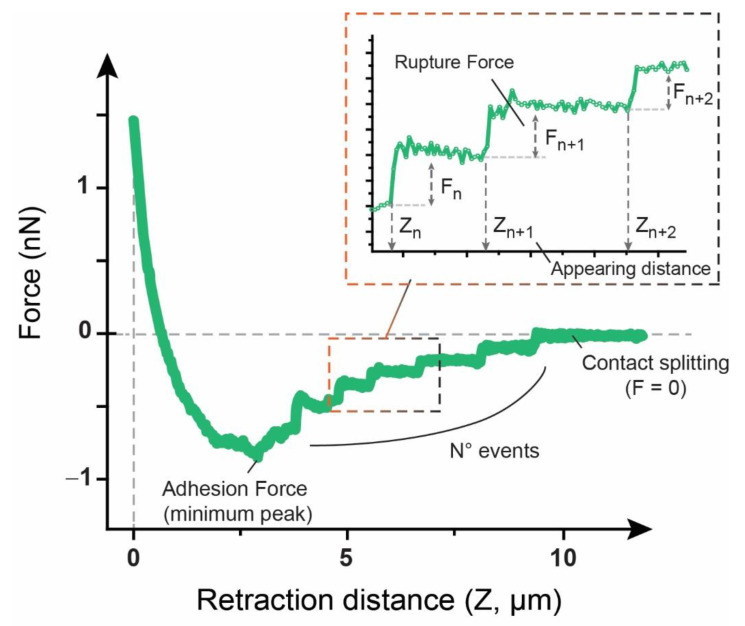
Representative force vs. distance plot from the retraction segment, including a detailed analysis of the different adhesion factors in it.

Adhesion Force was extracted from the minimum in the retraction plot (see Figure 1). Rupture events were determined using the stepwise recovery of the force after the minimum point, as shown in Figure 2, and their distance of appearance (Z_n_) and rupture force (F_n_, step height) were individually considered and plotted.

## 3. Results

Indentation of the CRC cell line HT-29-MTX allowed for extracting mechanics-related information from each of the different segments; the force-distance curves were composed of approach, relaxation, and retraction. A descriptive analysis of these individual components is necessary to understand the time dependence of factors such as the elastic (Young’s) modulus, relaxation time, and adhesion force, and to evaluate the cell behavior using membrane pulling. In turn, a joint consideration of all of these components can help with drawing an overall picture of the mechanical response at the nanoscale, where the existence of correlative trends could be identified.

### 3.1. Approach and Pause: Elastic Modulus Determination and Stress Relaxation

**Figure 3:**
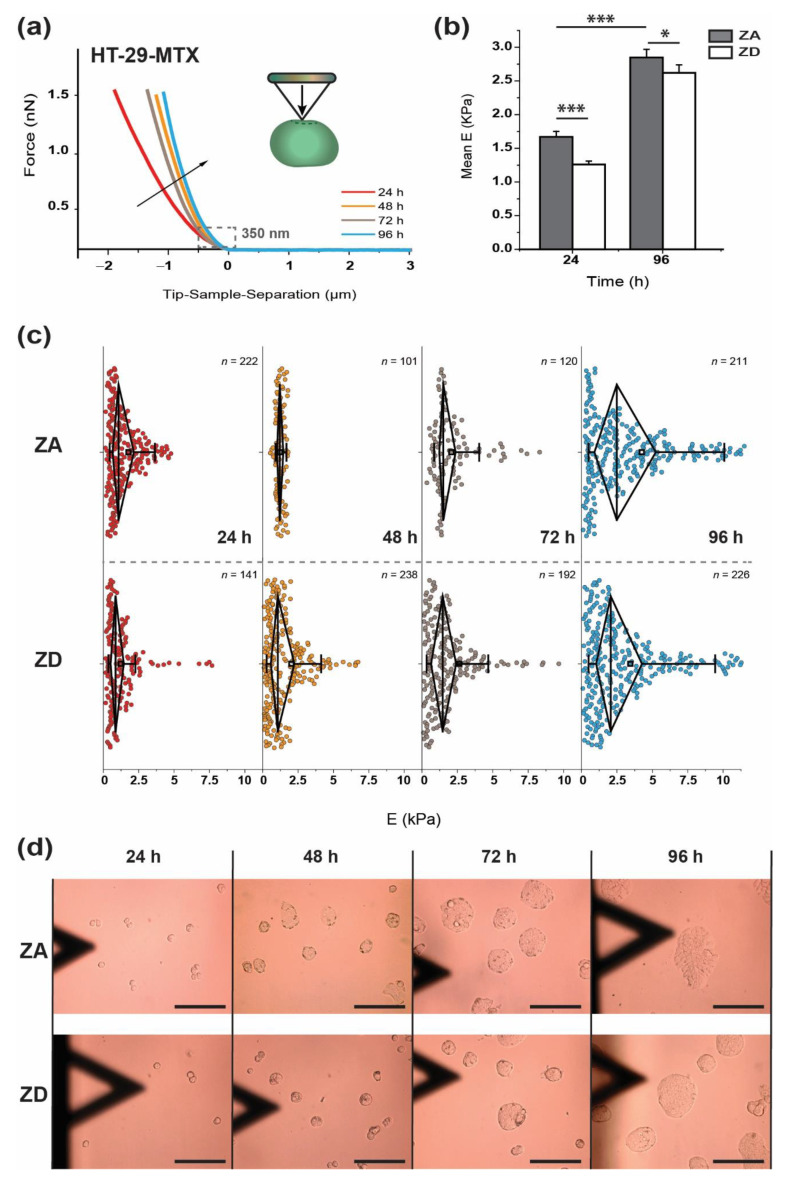
Time-dependent mechanical and morphological properties of HT-29-MTX cells. (**a**) Comparison of the average approach curves for each of the incubation times of HT-29-MTX (*n* > 150). The inset provides a schematic view of the tip motion during indentation. (**b**) Mean elastic modulus values ± standard error of mean (SEM). Filled columns correspond to zinc-adequate (ZA) cells while open columns correspond to zinc-deficient (ZD). Significant differences between ZA and ZD cells are indicated (* *p* < 0.05; *** *p* < 0.001; Student *t*-test). (**c**) Distribution of Young’s modulus individual values. Black diamond-shaped boxes indicate the 25–75% range of the distribution, with the vertical line showing the median. Left and right whiskers indicate achievement of both the 5% and 95% ranges, respectively. (**d**) Micrographs showing the time evolution of the cellular aggregate size for HT-29-MTX cells in both ZA and ZD conditions. The triangular shadow is caused by the presence of the AFM cantilever. The scale bar corresponds to 50 µm (This individual figure can be found, with a larger magnification, in the Figure A2).

Figure 3 depicts the evolution of mechanical and morphological properties of HT-29-MTX cells with the incubation time, either in the presence (zinc-adequate, ZA) or in the absence of zinc (zinc-deficient, ZD), when focusing on the approach segment. A comparison between the average approach force-distance plots obtained for different incubation times offers a quick distinction between the respective cell states: The variation in slope indicates changes of cell stiffness (in nN/µm) under the identical approach rate and maximum load conditions. In order to quantify the differences in membrane compressibility, the initial 350 nm of the indentation plots was fitted with a Hertz-Sneddon model (see Equation (2)).

**Figure A1:**
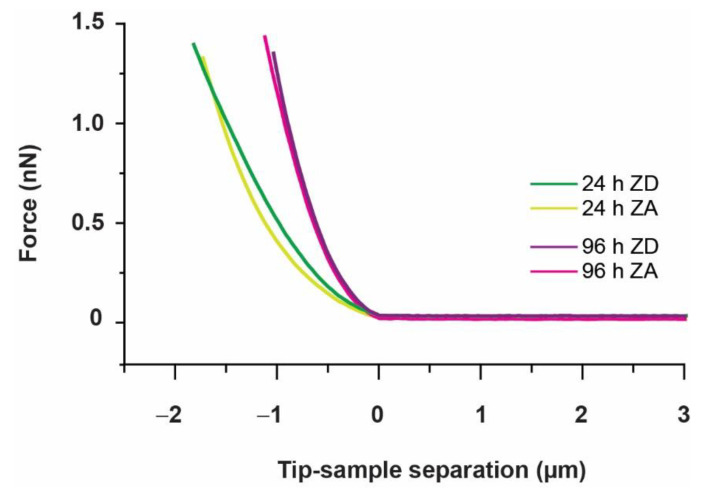
Average approach plots for HT-29-MTX cells at two time points (24 and 96 h), and different zinc exposure conditions.

Both ZA and ZD HT-29-MTX cells appeared to be stiffer over an ongoing cultivation time. A similar variation between ZA and ZD based on their respective approach plots at 24 and 96 h can be seen (Figure A1). The average Young’s modulus values for ZA cells at these time points are 30% and 20% higher than for the ZD ones (Figure 3b). Accordingly, cells in the presence of zinc were less deformable (or stiffer) at the beginning of cell cultivation (24 h), and gradually resembled the behavior of ZD cells with progressing proliferation. In addition to the mean values, distribution of E values illustrates heterogeneity found in the mechanical response of the cultures (Figure 3b,c).

After 24 h of incubation, cells offered a very compact distribution of the individual data (despite the presence of a few outliers), which gradually split into different populations over an ongoing incubation time. For the longest incubation time, the evident spreading in the elastic modulus data could have been related to the increasing size of two-dimensionally growing aggregates (see Table 1), which induce the stiffening of a larger number of cells (as represented in Figure 3d). Additionally, a larger number of soft HT-29-MTX cells was identified. Cell indentation was performed, for all the conditions tested, in the outermost region of cell aggregates. There, cells suffer less from the influence of neighboring cells, in comparison with the more restricted situation at the inner part of that region. However, as these aggregates evolved, a larger number of cells from the peripheral positions also start to be affected by their environment, and this might cause the presence of two well-defined cell groups with differing stiffness/softness. Then, the softening effect occurs almost identically in both ZA and ZD HT-29-MTX cells, although cells cultured in the presence of zinc seem to develop such behavior with a delay in time compared to ZD cells. Indeed, ZA samples that are measured after 48 and 72 h show a quite narrow distribution around intermediate E values. When extending the incubation to 96 h, the population of E values observed below the average value is significantly larger in ZD cells, considering that the number of samples tested is almost equal (*n* = 211 vs. *n* = 226). The shifting of the median toward lower values indicates that the presence/absence of zinc has a strong impact on cellular development and mechanics.

**Figure A2:**
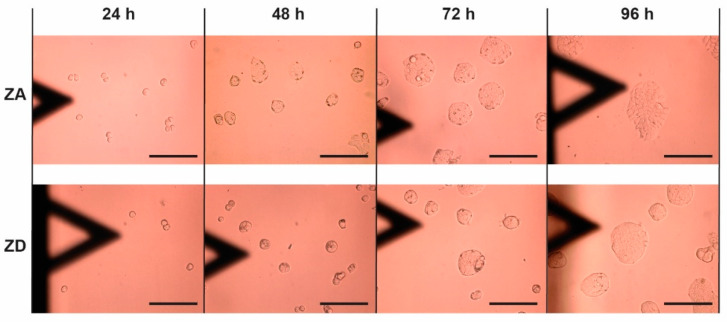
Micrographs showing HT-29-MTX cell aggregation over time, under both ZA and ZD conditions. The triangular shadow is caused by the presence of the AFM cantilever. The scale bar corresponds to 50 µm.

**Figure A3:**
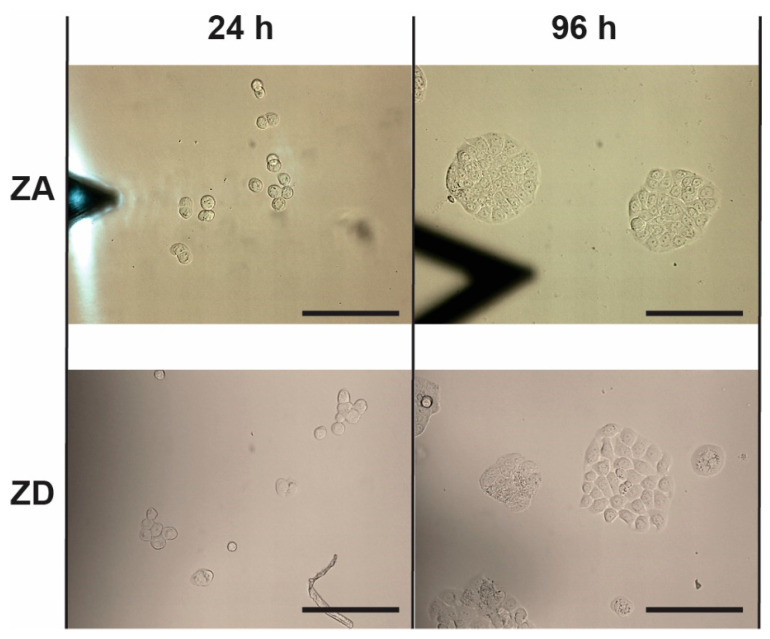
Micrographs showing HT-29 cell aggregation over time, under both ZA and ZD conditions. The triangular shadow is caused by the presence of the AFM cantilever. The scale bar corresponds to 50 µm.

To further investigate the impact of cultivation time and zinc-supply on the deformability of CRC cells, elastic moduli of HT-29 were measured, and the mean Young’s moduli were compared to those calculated for HT-29-MTX, as this can be considered a good indicator of existing differences between these cell lines (Table 2). The morphological variations of HT-29 cells in this period, and the size of the appearing aggregates were also controlled by using optical microscopy (Figure A2 and Figure A3). For HT-29 cells, the comparison was kept only for 24 and 96 h time points, since they showed the most extreme values and the influence of zinc could be more easily observed.

**Figure 4:**
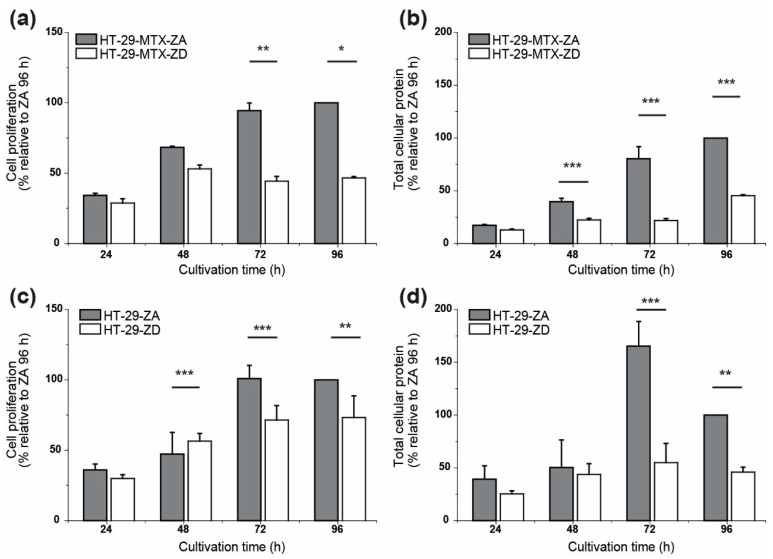
Impact of zinc-deficient cultivation on cell proliferation of HT-29-MTX (**a**,**b**) and HT-29 (**c**,**d**) cells. Metabolic activity of cells (**a**,**c**) grown for 24–96 h in either zinc-adequate (ZA) or -deficient (ZD) medium, was measured with water soluble tetrazolium (WST), and total cellular protein (**b**,**d**) was determined using SRB. Significant differences between ZA and ZD cells are indicated (* *p* < 0.05; ** *p* < 0.01; *** *p* < 0.001; Two-Way ANOVA with Bonferroni post-hoc test), as are means + SD of three independent experiments.

The time-dependent increase of the calculated elastic modulus was stronger for HT-29-MTX than for HT-29 cells, which retained values of around 1.0 KPa over the same period. After 96 h of incubation HT-29-MTX cells presented a 4- to 5-fold larger elastic modulus than HT-29. There also appeared to be a variation between ZA and ZD HT-29 systems after 96 h (ca. 35%) in a similar manner to what was observed for HT-29-MTX (a drop of 20%, Figure 3b). Thus, a deficiency of zinc led to softer cells in both cell lines. In terms of aggregate formation, HT-29 grew rather separately with a tendency for monolayer formation (after 96 h) (Figure A3). Indeed, HT-29 cells showed very similar sizes after 24 h, with more remarkable variations after 96 h of incubation. In comparison with HT-29-MTX, aggregates formed by HT-29 after 96 h (under both ZA and ZD conditions) were 30–40% smaller in size. This also reflects the impact of zinc in cell proliferation (Figure 4), as the lack of this essential trace element significantly impairs cell proliferation, measured as changes in metabolic activity and total cellular protein over time, which leads to diminished cell growth and density. Cell proliferation of ZD HT-29-MTX seemed to cease after 48 h of cultivation, only providing 50% proliferating cells compared to ZA cells (Figure 4a), whereas proliferation of ZA and ZD HT-29 cells both increased up to 72 h, with only 20% less metabolic activity of zinc-depleted cells (Figure 4c). The impact of zinc deprivation on total protein content was predominantly present for HT-29-MTX, showing a very slight increase of cellular protein that reached 50% of the cellular protein content of ZA cells after 4 days of cultivation (Figure 4b). Likewise, ZD HT-29 cells contained 50% less protein than ZA cells, yet the effect of zinc on cell growth was only visible after zinc depleted cultivation for 72 h (Figure 4d).

**Figure 5:**
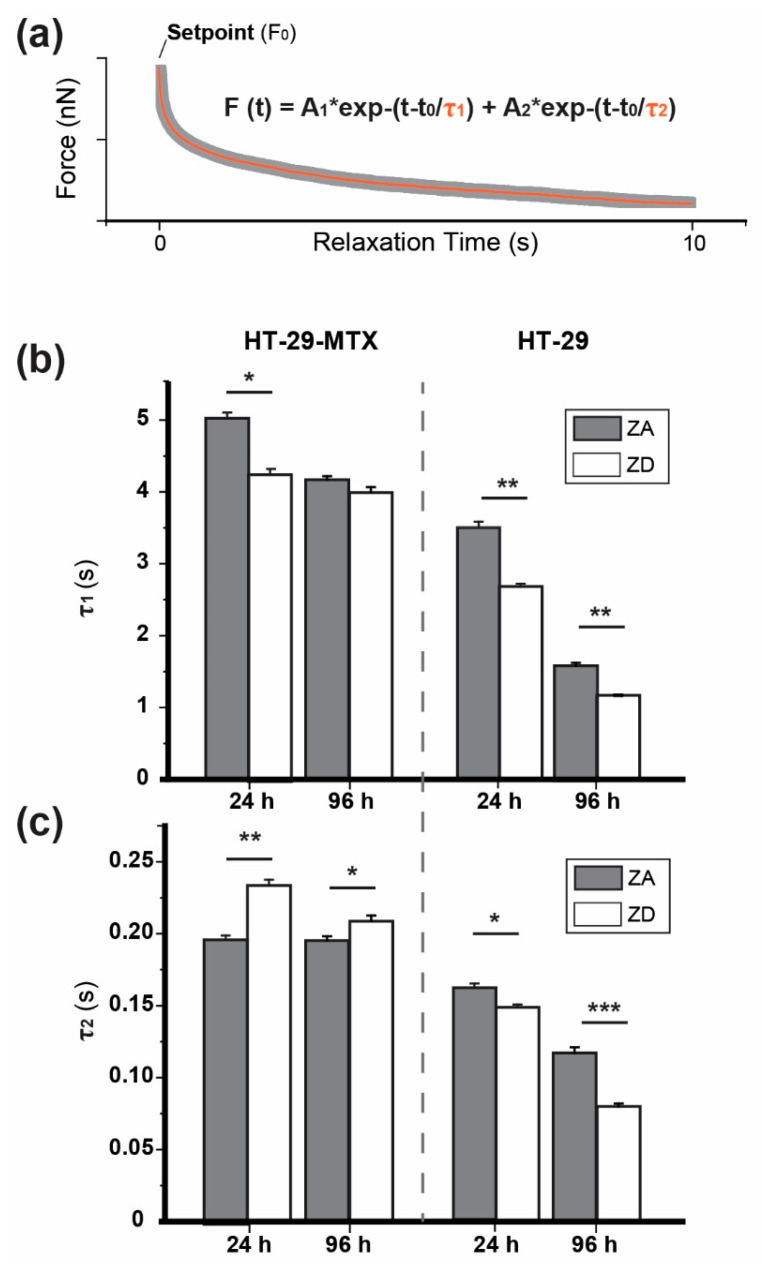
Stress relaxation profile analysis. (**a**) Pause segment showing the stress relaxation path followed and the double-exponential curve fitting. Below, a comparison of calculated τ_1_ (**b**) and τ_2_ (**c**) values for HT-29-MTX cells and HT-29 cells cultivated in the presence (ZA) or absence (ZD) of zinc for 24 or 96 h is shown (*n* > 50). Error bars correspond to the Standard Error of the Mean. Significant differences, as determined by Student’s *t*-test, are indicated (* *p* < 0.05; ** *p* < 0.01; *** *p* < 0.001).

In a second step of calculation, as the maximum indenting load is reached, the tip was kept in contact with the cell for 10 s by pre-defining the fixed position of the cantilever in Z (height), and thus letting the cell undergo a relaxation process. This allowed the in-situ calculation of the stress relaxation of these cells. In this case, the stress decay plot—which is typical of non-elastic bodies—can be quite accurately fitted with a time-dependent double exponential, as previously described by Moreno–Flores et al. [64] (Figure 5): At *t* = 0 s, the contact time started and the initial force (maximum load or setpoint, F_0_) decreased over the observation time. For sufficiently long contact times, the force could have even reached stabilization. From the fitting equation, τ_1_ and τ_2_ relate to the relaxation time of two different elements, where the longer one (τ_1_) was connected to the overall cytoskeleton response, while τ_2_ possibly refers to the membrane response. Table 3 collects τ_1_ and τ_2_ values calculated from the respective fittings (including their goodness) and measuring conditions.

In case of HT-29-MTX, τ_1_ and τ_2_ values remained almost unaltered when culturing cells w/o zinc for 24 h or 96 h, apart from the slight drop (<10%) for ZA cells with progressing incubation (24 vs. 96 h), which was very similar to that between ZA and ZD cells at 24 h. In contrast, calculated relaxation time values of HT-29 decreased sharply by 55% and 60% with the incubation time, possibly reflecting the gradual increase of cell numbers and density during proliferation. This was indicated by the drop in both τ_1_ (ZA: 3.51 s to 1.60 s; ZD: 2.68 s to 1.13 s) and τ_2_ (ZA: 0.16 s to 0.12 s; ZD: 0.15 s to 0.08 s). In summary, the relaxation process showed that the individual cellular properties of the cancerous cell line predominate over the zinc supply, particularly for ZD CRC cell line HT-29.

### 3.2. Adhesion and Rupture Events

As relaxation time reached its end, the tip was immediately pulled away at a constant speed (5 µm/s), and the resulting force variation taking place as retraction occurs was monitored (Figure 2). Such variation will depend on the affinity the tip has for the cell and, in addition, on the degree of connection between the cortex and the cell membrane, which influences the accessibility of the latter.

**Figure A5:**
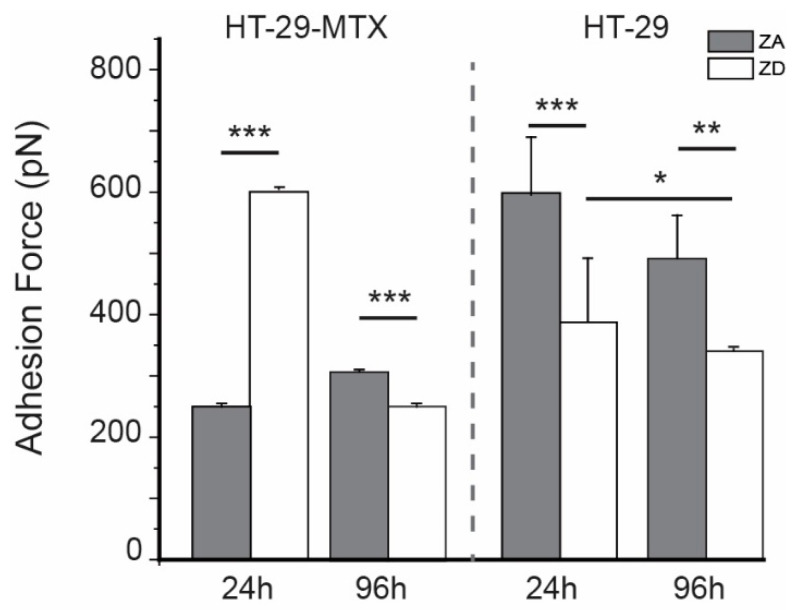
Mean adhesion force values for both HT-29-MTX and HT-29 cells (*n* > 50) in both ZA and ZD conditions. Error values correspond to the standard error of mean (SEM). Significant differences, as determined by Student’s *t*-test, are indicated (* *p* < 0.05; ** *p* < 0.01; *** *p* < 0.001;).

The minimum in the retraction plot is related to the maximum force that has to be applied in order to split the contact between the tip and the indented cell. The obtained average values are presented in Table 4 (see also Figure A5). As the indenting tip employed had no particular coating causing its specific recognition, the type of tip-cell contact is considered merely non-specific. Therefore, adhesion forces remain within lower ranges than for specific interactions.

HT-29-MTX cells incubated under ZD conditions for 96 h showed remarkable changes, with a drop in the maximum adhesion of around 60% down to 240 pN. This lower adhesion was similar to that from HT-29-MTX cultivated in the presence of zinc as well as ZD HT-29, and was comparable to other cell lines measured previously under similar conditions (MCF7, Caco-2) [18,24]. The mean adhesion force of ZA HT-29 cultivated for 96 h, however, was twice as high as that for ZA HT-29-MTX at the same time-point.

**Figure 6:**
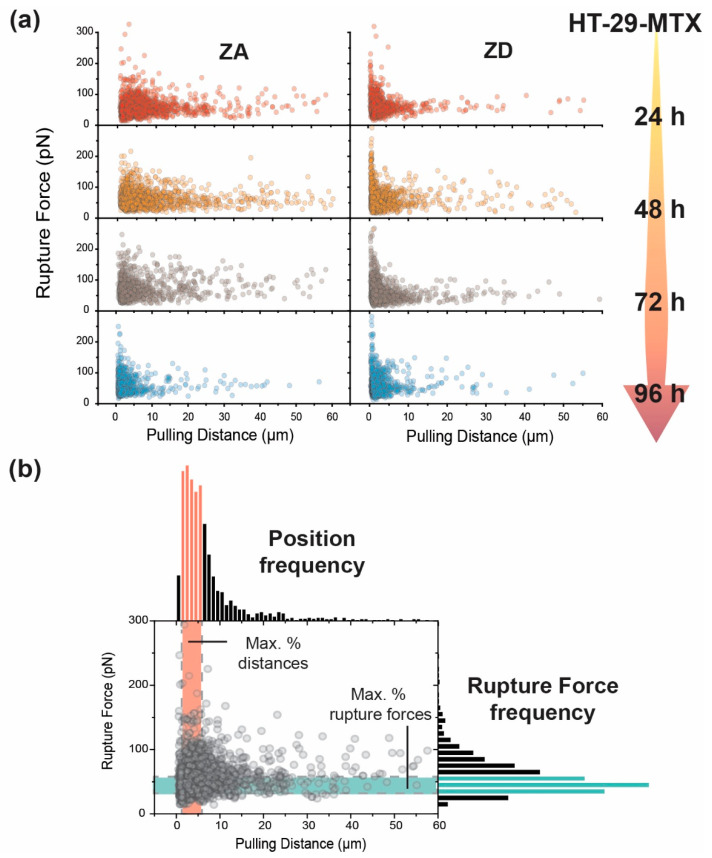
Rupture event fingerprint characterization, as obtained from membrane pulling during tip retraction. (**a**) Time and zinc exposure dependent rupture event distribution (*n* > 1000) for HT-29-MTX cells. The colored arrow indicates the direction of the incubation time elapsed. (**b**) Combined histogram presentation of Rupture Force (in pN) and retraction distance (in µm) factors, highlighting the ranges of a higher probability of events on each axis.

Hence, a detailed analysis was performed on the full set of rupture events taking place before the tip and the cell membrane could fully unhook, and the force could recover its zero value. A comparison can be obtained by analyzing the distribution of individual events and plotting their rupture force against distance of appearance (Figure 6). It can be seen rather clearly how the pattern followed by the dotted distribution varied as the cultivation time increased, and how much these distributions are influenced by either the presence or the absence of zinc. Due to the large amount of data plotted, and in order to achieve a better visualization and obtain descriptive information, the respective histogram distribution on each axis is included (Figure 6b). Through consideration of the 3–5 most probable bins (colored bands), the areas of highest point density were defined.

**Figure 7:**
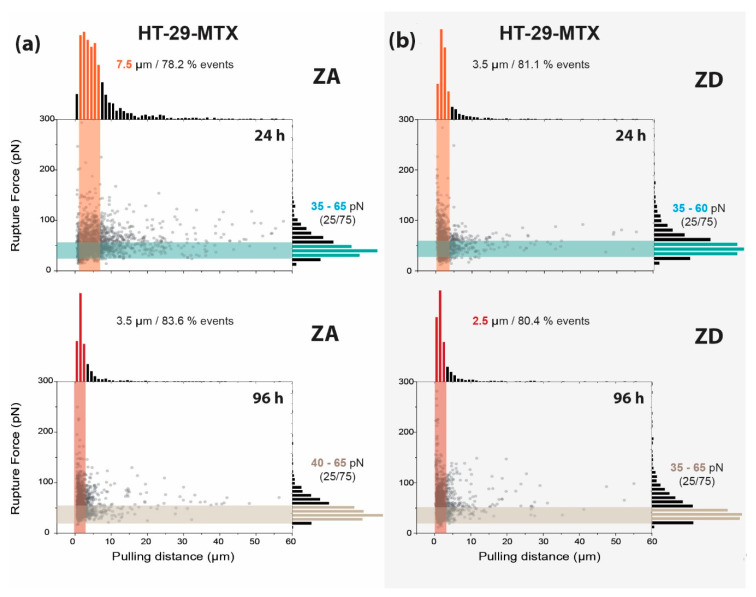
Influence of zinc on the rupture event fingerprint of HT-29-MTX cells. Rupture event and histogram distribution combination for zinc-adequate (ZA) (**a**) and zinc-deficient (ZD) (**b**) HT-29-MTX cells after incubation for 24 and 96 h (*n* > 1000). The rupture force range indicated on the right corresponds to the 25–75% range of the events.

Rupture events after 24 h presented a noticeable broader distribution for ZA HT-29-MTX (Figure 7a, top) where the range of the pulling distance was extended up to 7.5 µm to visualize around 80% of ruptures. For ZD cells, the same percentage of events was observed after only 3.5 µm (Figure 7b, top). Above a pulling distance of 10 µm, ZD HT-29-MTX showed very few events (<5% of data), while their zinc-sufficient counterpart exhibited around 15% of events. In the latter, the number of ruptures appeared to be quite regularly distributed, with a logical decrease as the pulling distance increased. After 96 h, both systems showed a tendency to narrow their event distribution. This trend was more remarkable for ZA cells, resembling the results obtained after zinc restriction for 24 h (83% of the events at 3.5 µm), but could also be observed for ZD cells after 96 h, where 80% of the ruptures already appeared below 2.5 µm.

In terms of rupture forces, it is quite surprising that these remained centered around similar values (35–65 pN) in HT-29-MTX cells, independently from their exposure to zinc, the incubation time, and the distance at which the ruptures appeared. Moreover, at short pulling distances, the probabilities of measuring larger forces were certainly higher in all of the cases.

**Figure 8:**
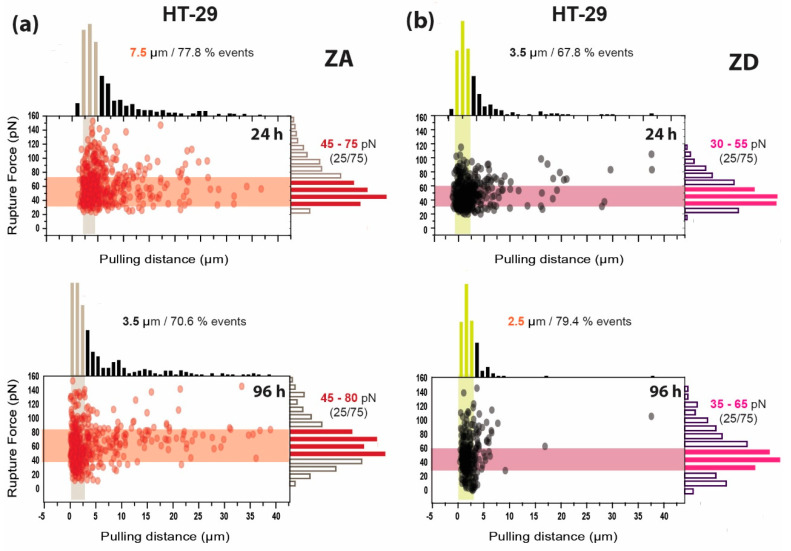
Influence of zinc on the rupture event fingerprint of HT-29 cells. Rupture event and histogram distribution combination for zinc-adequate (ZA) (**a**) and -deficient (ZD) (**b**) HT-29 cells after incubation for 24 and 96 h (*n* > 300). The rupture force range indicated on the right corresponds to the 25–75% range of the events.

When comparing these results to those obtained for HT-29 cells (Figure 8) under the same experimental conditions, rupture forces on ZA HT-29 cells appeared to be larger (45–80 pN) than those reported above, while ZD cells remained close to the values shown by HT-29-MTX samples (30–65 pN). Then, the zinc supply considerably affects the membrane accessibility of HT-29 cells. Also, the value for the pulling distance required for reaching a percentage of events of around 80% for zinc abundant HT-29 cells increased up to around 8.5 µm after 96 h, in comparison with the 3.5 µm needed for HT-29-MTX. However, for ZD HT-29 cells these values resembled those of HT-29-MTX.

**Figure 9:**
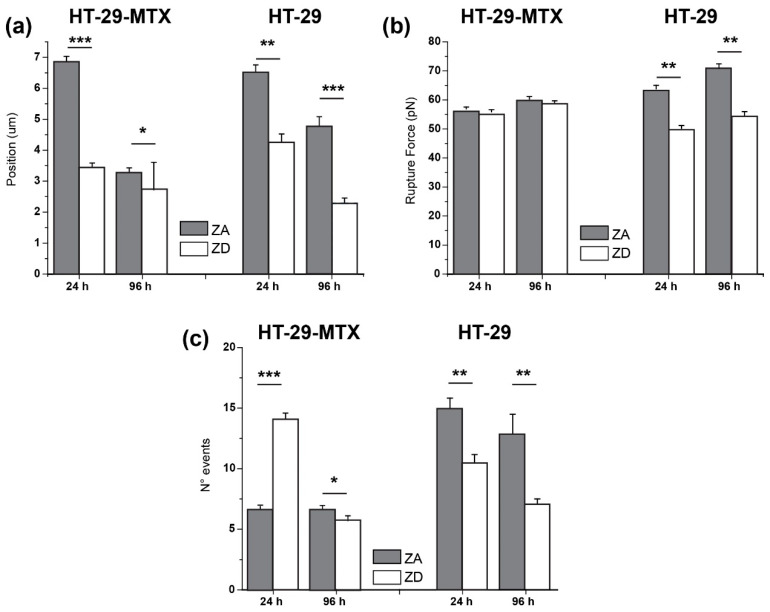
Adhesion factors associated with the rupture events recorded from pulling HT-29-MTX (empty boxes) and HT-29 cells (filled boxes). (**a**): Event appearing position; (**b**): Rupture Force; (**c**): Number of rupture events; Means ± standard error of mean (SEM) (*n* > 50) are indicated. Significant differences between ZA and ZD cells, as determined by Student’s *t*-test, are indicated (* *p* < 0.05; ** *p* < 0.01; *** *p* < 0.001).

For better quantification and comparison between the two cell lines and incubation conditions, the calculated average pulling positions and rupture forces, together with the average number of events taking place per experiment, are depicted in Figure 9 (corresponding data in Table 5). These values illustrate how time- and zinc-dependent variations take place: for ZD HT-29-MTX, the trend rather precisely resembled that of HT-29, and incubation time seemed to be the determining variable within the three considered factors. Yet, the cell line seemed to influence the amount of events, as the number of events produced by ZA HT-29-MTX were considerably lower than those of ZA HT-29 and increased sharply when depriving cells of zinc, whereas the number of events measured for ZD HT-29 cells decreased.

## 4. Discussion

By examining the CRC cell lines HT-29 and HT-29-MTX with AFM under adequate and deficient nutritional zinc supply, this study demonstrates that the nanomechanical properties of proliferating CRC cells are dependent on their zinc status. Of note, the application of zinc-depleted medium is a standardized method to subject cells to zinc deficiency in vitro [58], whereas the zinc content of zinc-adequate culture medium is in the same magnitude as the total zinc levels in human serum [65]. Even though data in the literature is not yet fully conclusive, there is increasing evidence that individual zinc status is related to CRC risk [37], thus differences in the zinc status of healthy tissue and tumorous tissue can occur and have to be taken into account when monitoring the biomechanical properties of cancerous cells. Among these parameters, the elasticity of cells is discussed to be the most suitable biomarker for cancer [66]. Estimated elastic moduli in this study are in the same order of magnitude as expected for eukaryotic cells, varying between several hundred Pascal to 10 kPa [3,25], and are comparable to previous studies on cancerous cells with different tissue origins [8]. Yet, comparison of absolute Young’s moduli determined in different studies is known to be difficult, as its measurement is highly dependent on several factors such as tip geometry and coating, as well as cell experimental conditions [8]. The sharp increase and heterogeneity of the calculated elastic moduli during cell proliferation of HT-29-MTX reflect the tendency of this CRC cell line to form aggregates [67], as cells become stiffer with elevated cell density [8]. Yet, the rise of the Young’s modulus during the aggregate formation of cells seems to be dependent on the cell line, as the elasticity of prostate cancer cell line PC-3 only changed very slowly with an increased number of neighboring cells [8], which was comparable to the estimated small changes for HT-29. In the absence of zinc, the elasticity and deformability of both CRC cell lines increased, leading to softer cells with lower cell density, which was also illustrated by their decreased protein content and metabolic activity. Similar to previous studies with ZD rat colonocytes [68] as well as pre- and post-confluent HT-29-MTX cells [58], the lack of zinc already significantly altered cell proliferation of both cell lines in early pre-confluent states. Likewise, zinc deficiency affected the measured rupture events, diminishing the rupture force, particularly for ZD HT-29, the number of events, as well as the event position. This could indicate that the membrane of ZD cells is less accessible for the tip, which could be due to the presence of a tighter connection of the membrane with the cytoskeleton. Similar behavior was already observed in other cell lines under exposure to environmental modifications and drugs [18]. Hence, the biomechanics of CRC were severely affected by the lack of zinc, and started to become softer and increasingly deformable with prolonged zinc deprivation. As zinc is essential for many biological functions in the human body, such as cell growth, differentiation, and apoptosis, and is required for numerous (metallo-) proteins for catalytic, regulatory, and structural functions [27], the consequence that the lack of this micronutrient also crucially impacts cellular biomechanics is not farfetched. Apart from the observed altered cell proliferation and aggregate formation, differences in their protein composition during zinc deficiency, particularly their cytoskeleton, cell-cell adhesion, and junctional proteins, might further explain these cell mechanical changes. While zinc deprived Caco-2 enterocytes revealed diminished expression of junctional and cytoskeletal proteins [49], zinc addition reportedly modifies their tight junction formation [69]. This was also shown in differentiating HT-29 colonocytes, where zinc chelation decreased the expression of the cell-adhesion protein E-cadherin, as well as the junctional proteins occludin and zonula occludens (ZO)-1 [70] and deregulated genes associated with cytoskeleton and cell-cell interaction on the transcriptional level [59]. Zinc regulates proliferation and growth of CRC cells by modulating the extra cellular signal regulated kinase (ERK) pathway [71,72,73]. Additionally, the β-catenin/WNT signaling pathway, which plays a regulatory role in CRC tumorigenesis and expression of proteins relevant for cell-cell junction and adhesion proteins [74], was shown to be zinc-dependently regulated, being either activated by zinc in osteosarcoma [75] or impaired in zinc-deficient neuronal stem cells [76]. Yet, it has to be noted that this study aimed to examine the impact of zinc and its deficiency on the mechanical response of proliferating CRC cells using AFM as a descriptive and monitoring tool. The degree to which these proteins are affected in proliferating zinc-deprived HT-29 and HT-29-MTX, and the degree to which their deregulation could be correlated with the changed biomechanics of these two CRC cell lines, both remain to be investigated.

Even though the cell mechanics of both HT-29 and HT-29-MTX were affected by zinc deficiency, their biomechanics, particularly their average elasticity, relaxation events, and adhesion factors, differed when studied under zinc adequate conditions. Analysis of elastic moduli and stress relaxation demonstrated that ZA HT-29 cells are softer than HT-29-MTX, while also resulting in a higher rupture force and number of adhesion events. To find a connection between the measured adhesion factors and cell stiffness, one might argue that the softer the cell is, the larger the rupture forces are, as well as the higher the number of events observed. This hypothesis however only matches with ZA samples, whereas the studied adhesion factors of ZD cells instead resemble the behavior of HT-29-MTX. Differences between the two CRC cell lines could be caused by their differing mechanical responses to the elevated cell density, as well as their individual cellular properties determined by their differing phenotype and cell morphology. Even though HT-29 cells are known to form multi-layers when reaching post-confluency after cultivation for 30 days under standard conditions [52], proliferating HT-29 grow rather separately with a tendency towards monolayer formation. The HT-29-MTX clone used in this study, HT-29-MTX-E12, was originally selected by Behrens et al. based on its ability to build mono-layers during its differentiation after 14–21 days of culture [56], and started to form aggregates after 48 h of proliferation. Increased cell density and cell-cell contacts elevates cell stiffness [8], which explains the decreased deformability and elasticity of ZA HT-29-MTX with ongoing cell proliferation as well as the slower response of HT-29, as already discussed. Moreover, this homogenous HT-29-MTX cell line originated from a sub-population of proliferating HT-29 cells [56]. Being capable of differentiating into mucin-producing cells upon reaching confluence after 7 days of cultivation [57], HT-29-MTX are commonly used as a human in vitro goblet cell model [31]. In contrast, HT-29 colonocytes are rather heterogeneous and only contain a fraction of 0.5% goblet cells and can differentiate into mature intestinal cells when cultured under specific conditions [54,55]. Even though this study focuses on the very beginning of cell proliferation (24–96 h), differences in their mechanical response are already measurable, indicating that their cellular behavior and possibly their phenotype and morphology might already differ in their pre-confluent state. Consequently, their individual cell surface and (tight) junctional, and cytoskeletal protein composition might also already differ in their proliferative state and thereby impact their cell adhesion, cell-cell contact, and mechanical cell properties, which all influence their cellular mechanical response. The differing biomechanics of the two CRC cell lines emphasize their relevance for comprehensively screening various CRC cells [77] by using AFM to identify and characterize nanomechanical fingerprints for tumor diagnostics. This requires using standardized and reproducible experimental conditions [66], as well as considering the impact of the extracellular matrix of CRC biopsies [21] on their mechanical properties, which needs to be incorporated into the identification and validation process of biomechanical markers for CRC. To distinguish between the impact of individual cancer and of zinc status on the mechanical behavior of cells, it is important to additionally screen zinc-adequate colon cells from non-pathological colon tissues, which can be achieved using primary colon cells [78]. Additionally, future studies of healthy and tumorous tissues via AFM should correlate results to the trace element status of the sample or patient, respectively, to monitor and further elucidate the impact of mineral malnutrition, particularly zinc deficiency, on the cellular nanomechanics of CRC.

## 5. Conclusions

This study provides insights into zinc-related and time-dependent biomechanical properties of proliferating CRC cells, which will help to further identify characteristic nanomechanical changes of cancerous cells as diagnostic biomarkers for CRC. Further comprehensive studies are needed to elucidate the underlying cellular processes on the transcriptional and protein synthesis/processing level and explain the observed changes in the mechanical response of proliferating HT-29 and HT-29-MTX colonocytes during zinc-deprivation. Moreover, the impact of zinc deficiency on the mechanical response of the cells underlines the relevance of monitoring the nutritional zinc status of tumor samples when analyzing cancerous tissues or single cells with AFM, particularly in the context of the development and validation of biomechanical fingerprints as diagnostic markers for cancer.

## Figures and Tables

**Table 1:** Mean time-dependent aggregate size comparison for HT-29-MTX and HT-29 cells (*n* > 20). The error deviation corresponds to the Standard Error of the Mean (SEM). See also Figure A4 to visualize the related statistically significant variations.

Incubation	HT-29-MTX		HT-29	
	ZA	ZD	ZA	ZD
24 h	210.7 ± 14.7 µm^2^	111.0 ± 9.6 µm^2^	203.7 ± 26.2 µm^2^	231.3 ± 71.2 µm^2^
48 h	648.9 ± 54.2 µm^2^	334.8 ± 25.6 µm^2^		
72 h	1721.8 ± 227.2 µm^2^	751.2 ± 100.1 µm^2^		
96 h	3390.9 ± 526.4 µm^2^	3108.1 ± 344.2 µm^2^	2286 ± 571.7 µm^2^	2064.9 ± 923.5 µm^2^

**Table 2:** Mean elastic (Young’s) moduli comparison for HT-29-MTX and HT-29 cells (*n* > 50) at two different time points (24 and 96 h). The error deviation corresponds to the Standard Error of the Mean (SEM). Table A1 collects the entire set of mean Young’s modulus values. Statistically significant variations are shown in Figure 3b.

Incubation	HT-29-MTX		HT-29	
	ZA	ZD	ZA	ZD
24 h	1.67 ± 0.08 kPa	1.26 ± 0.05 kPa	0.91 ± 0.05 kPa	1.09 ± 0.06 kPa
96 h	2.85 ± 0.28 kPa	2.62 ± 0.12 kPa	1.28 ± 0.17 kPa	0.85 ± 0.08 kPa

**Table 3:** Mean τ_1_ and τ_2_ values (*n* > 50) for both HT-29-MTX and HT-29 cells after 24 and 96 h of incubation either in the presence (ZA) or absence of zinc (ZD). The goodness of the double exponential fitting is represented by a r square (r^2^) factor. Error values correspond to the standard error of mean (SEM). See Table A2 for the entire set of mean relaxation times.

Incubation	HT-29-MTX		HT-29	
	ZA	ZD	ZA	ZD
24 h	τ_1_ = 5.02 ± 0.08 s	τ_1_ = 4.19 ± 0.06 s	τ_1_ = 3.51 ± 0.08 s	τ_1_ = 2.68 ± 0.04 s
	τ_2_ = 0.19 ± 0.003 s	τ_2_ = 0.23 ± 0.003 s	τ_2_ = 0.16 ± 0.003 s	τ_2_ = 0.15 ± 0.002 s
	r^2^ = 0.9950	r^2^ = 0.9958	r^2^ = 0.9964	r^2^ = 0.9976
96 h	τ_1_ = 4.10 ± 0.05 s	τ_1_ = 3.99 ± 0.06 s	τ_1_ = 1.60 ± 0.08 s	τ_1_ = 1.13 ± 0.01 s
	τ_2_ = 0.19 ± 0.004 s	τ_2_ = 0.21 ± 0.004 s	τ_2_ = 0.12 ± 0.004 s	τ_2_ = 0.08 ± 0.002 s
	r^2^ = 0.9962	r^2^ = 0.9941	r^2^ = 0.9839	r^2^ = 0.9954

**Table 4:** Mean Adhesion Force values (*n* > 50) for both HT-29-MTX and HT-29 cells after 24 and 96 h of incubation either in the presence (ZA) or absence of zinc (ZD). Error values correspond to the standard error of mean (SEM). Table A3 shows the entire set of mean Adhesion Forces, while the statistical significances of the variations observed are depicted in Figure A5.

Incubation	HT-29-MTX		HT-29	
	ZA	ZD	ZA	ZD
24 h	0.29 ± 0.01 nN	0.59 ± 0.03 nN	0.71 ± 0.24 nN	0.38 ± 0.09 nN
96 h	0.31 ± 0.02 nN	0.24 ± 0.01 nN	0.60 ± 0.07 nN	0.33 ± 0.02 nN

**Table 5:** Mean event position and rupture force values for both HT-29-MTX and HT-29 cells at 24 and 96 h of incubation (*n* > 50). Error corresponds to the standard error of mean. See Table A4 for the entire set of event positions and rupture forces.

Incubation	HT-29-MTX		HT-29	
	ZA	ZD	ZA	ZD
	Mean Event Position			
24 h	6.86 ± 0.17 µm	3.48 ± 0.14 µm	6.49 ± 0.24 µm	4.33 ± 0.23 µm
96 h	3.23 ± 0.15 µm	2.69 ± 0.89 µm	4.76 ± 0.31 µm	2.28 ± 0.17 µm
	Mean Rupture Force			
24 h	56.9 ± 1.23 pN	55.9 ± 0.76 pN	63.8 ± 1.23 pN	50.2 ± 1.01 pN
96 h	60.3 ± 0.83 pN	58.8 ± 0.89 pN	70.9 ± 1.49 pN	54.3 ± 1.66 pN

**Figure A4:**
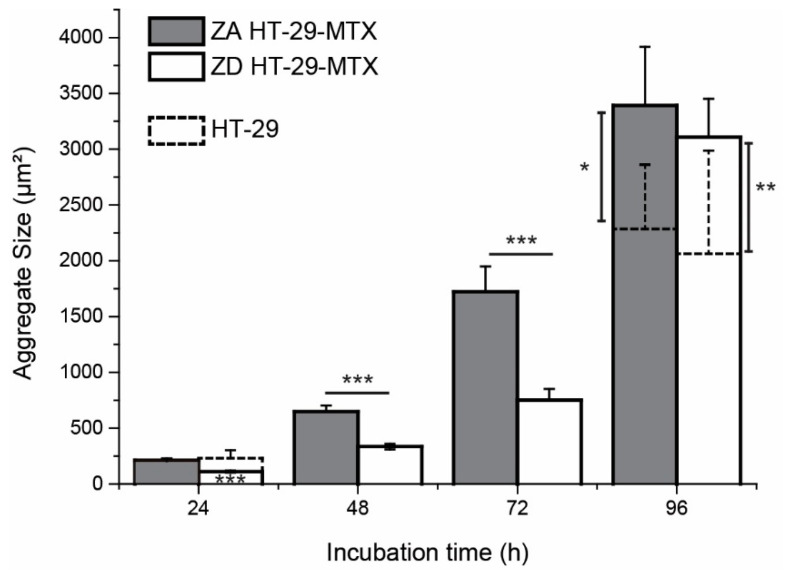
Mean time-dependent aggregate size comparison for HT-29-MTX and HT-29 cells (*n* > 20) in both ZA and ZD conditions. Error deviation corresponds to the Standard Error of the Mean (SEM). Significant differences, as determined by Student’s *t*-test, are indicated (* *p* < 0.05; ** *p* < 0.01; *** *p* < 0.001;).

## Appendix A

**Table A1:** Mean elastic (Young’s) moduli comparison for HT-29-MTX and HT-29 cells (*n* > 50) at different time points (24, 48, 72 and 96 h) in both ZA and ZD conditions. The error deviation corresponds to the Standard Error of the Mean (SEM).

Incubation	HT-29-MTX		HT-29	
	ZA	ZD	ZA	ZD
24 h	1.67 ± 0.08 kPa	1.26 ± 0.05 kPa	0.91 ± 0.05 kPa	1.09 ± 0.06 kPa
48 h	1.25 ± 0.03 kPa	1.37 ± 0.06 kPa		
72 h	1.58 ± 0.07 kPa	1.62 ± 0.08 kPa		
96 h	2.85 ± 0.12 kPa	2.62 ± 0.12 kPa	1.28 ± 0.17 kPa	0.85 ± 0.08 kPa

**Table A2:** Mean relaxation time comparison for HT-29-MTX and HT-29 cells (*n* > 50) at different time points (24, 48, 72 and 96 h) and in both ZA and ZD conditions. The error deviation corresponds to the Standard Error of the Mean (SEM).

Incubation	HT-29-MTX		HT-29	
	ZA	ZD	ZA	ZD
24 h	τ_1_ = 5.02 ± 0.08 s	τ_1_ = 4.19 ± 0.06 s	τ_1_ = 3.51 ± 0.08 s	τ_1_ = 2.68 ± 0.04 s
	τ_2_ = 0.19 ± 0.003 s	τ_2_ = 0.23 ± 0.003 s	τ_2_ = 0.16 ± 0.003 s	τ_2_ = 0.15 ± 0.002 s
	r^2^ = 0.9950	r^2^ = 0.9958	r^2^ = 0.9964	r^2^ = 0.9976
48 h	τ_1_ = 3.77 ± 0.05 s	τ_1_ = 4.33 ± 0.05 s		
	τ_2_ = 0.15 ± 0.004 s	τ_2_ = 0.16 ± 0.003 s		
	r^2^ = 0.9948	r^2^ = 0.9957		
72 h	τ_1_ = 5.83 ± 0.10 s	τ_1_ = 5.41 ± 0.12 s		
	τ_2_ = 0.24 ± 0.004 s	τ_2_ = 0.22 ± 0.005 s		
	r^2^ = 0.9958	r^2^ = 0.9919		
96 h	τ_1_ = 4.10 ± 0.05 s	τ_1_ = 3.99 ± 0.06 s	τ_1_ = 1.60 ± 0.08 s	τ_1_ = 1.13 ± 0.01 s
	τ_2_ = 0.19 ± 0.004 s	τ_2_ = 0.21 ± 0.004 s	τ_2_ = 0.12 ± 0.004 s	τ_2_ = 0.08 ± 0.002 s
	r^2^ = 0.9962	r^2^ = 0.9941	r^2^ = 0.9839	r^2^ = 0.9954

**Table A3:** Mean adhesion force comparison for HT-29-MTX and HT-29 cells (*n* > 50) at different time points (24, 48, 72 and 96 h) and in both ZA and ZD conditions. The error deviation corresponds to the Standard Error of the Mean (SEM).

Incubation	HT-29-MTX		HT-29	
	ZA	ZD	ZA	ZD
24 h	0.24 ± 0.02 nN	0.59 ± 0.03 nN	0.61 ± 0.03 nN	0.37 ± 0.02 nN
48 h	0.34 ± 0.01 nN	0.31 ± 0.01 nN		
72 h	0.44 ± 0.02 nN	0.39 ± 0.01 nN		
96 h	0.32 ± 0.01 nN	0.24 ± 0.01 nN	0.47 ± 0.07 nN	0.33 ± 0.02 nN

**Table A4:** Mean event position and rupture force values for HT-29-MTX and HT-29 cells (*n* > 50) at different time points (24, 48, 72 and 96 h) and in both ZA and ZD conditions. The error deviation corresponds to the Standard Error of the Mean (SEM).

Incubation	HT-29-MTX		HT-29	
	ZA	ZD	ZA	ZD
	Mean Event Position			
24 h	6.86 ± 0.17 µm	3.48 ± 0.14 µm	6.49 ± 0.24 µm	4.33 ± 0.23 µm
48 h	7.49 ± 0.22 µm	3.74 ± 0.16 µm		
72 h	7.23 ± 0.28 µm	3.73 ± 0.14 µm		
96 h	3.23 ± 0.15 µm	2.69 ± 0.89 µm	4.76 ± 0.31 µm	2.28 ± 0.17 µm
	Mean rupture Force			
24 h	56.9 ± 1.23 pN	55.9 ± 0.76 pN	63.8 ± 1.23 pN	50.2 ± 1.01 pN
48 h	61.9 ± 0.65 pN	59.4 ± 0.87 pN		
72 h	73.0 ± 0.99 pN	58.1 ± 0.84 pN		
96 h	60.3 ± 0.83 pN	58.8 ± 0.89 pN	70.9 ± 1.49 pN	54.3 ± 1.66 pN
